# First evidence for the antitumor activity of nanoliposomal irinotecan with 5-fluorouracil and folinic acid in metastatic biliary tract cancer

**DOI:** 10.1007/s00280-020-04094-0

**Published:** 2020-06-18

**Authors:** Hossein Taghizadeh, Matthias Unseld, Andreas Schmiderer, Angela Djanani, Klaus Wilthoner, Dieter Buchinger, Gerald W. Prager

**Affiliations:** 1 Clinical Division of Oncology, Department of Medicine I, Medical University of Vienna, Vienna, Austria; 2 Comprehensive Cancer Center, Vienna, Austria; 3 Clinical Division of Gastroenterology, Hepatology and Metabolism, Department of Internal Medicine I, Medical University Innsbruck, Innsbruck, Austria; 4 Clinical Division of Oncology, Department of Medicine I, Salzkammergut Klinikum, Vöcklabruck, Upper Austria Austria

**Keywords:** Biliary tract cancer, Chemotherapy, Nanoliposomal irinotecan, Progression-free survival, Overall survival

## Abstract

**Background:**

Therapeutic options are limited for advanced, metastatic biliary tract cancer. The pivotal NAPOLI-1 trial demonstrated the superior clinical benefit of nanoliposomal irinotecan (Nal-IRI) in gemcitabine-pretreated patients with metastatic pancreatic ductal adenocarcinoma; however, the antitumor activity of Nal-IRI in biliary tract cancer is unknown. This is the first report describing the efficacy of Nal-IRI in biliary tract cancer.

**Methods:**

In this multicenter retrospective cohort analysis, we identified patients with metastatic biliary tract adenocarcinoma who were treated with Nal-IRI in combination with 5-fluorouracil and folinic acid following tumor progression under standard therapy at one of the study centers between May 2016 and January 2019. We assessed disease control rate (DCR), progression-free survival (PFS), and overall survival (OS).

**Results:**

There were 14 patients; the median age at the time of diagnosis and the median age at the initiation of Nal-IRI were 59.3 and 60.0 years, respectively. Nal-IRI in combination with 5-fluorouracil and folinic acid was administered as second-, third-, fourth-, and fifth-line treatment in 6 (43%), 5 (36%), 2 (14%), and 1 (7%) patient with metastatic disease, respectively. The objective DCR with Nal-IRI was 50% (7/14 patients). Six patients (43%) had partial response, and one patient (7%) had stable disease. Progressive disease was observed in seven patients. The median PFS and median OS following Nal-IRI initiation were 10.6 and 24.1 months, respectively.

**Conclusions:**

This retrospective analysis provides the first evidence that Nal-IRI might exhibit a clinical meaningful antitumor activity in metastatic biliary tract cancer.

## Introduction

Biliary tract cancer (BTC) is a highly malignant and fatal cancer that arises from the biliary epithelium of the bile duct, gallbladder, and the ampulla of Vater and encompasses several entities, including gallbladder carcinoma, extrahepatic cholangiocarcinoma (CCC), perihilar CCC, intrahepatic CCC, and ampullary carcinoma [1, 2]. BTC is an orphan disease with an incidence of about 2/100,000 [3]. Systemic chemotherapy is the only recommended treatment approach in patients with stage IV BTC [4], whereas the currently established first-line treatment for metastatic BTC includes gemcitabine in combination with cisplatin as suggested by the phase III ABC-02 trial (NCT00262769) [5]. Currently, there are no established second-line treatment protocols. In May 2019, Lamarca et al. introduced the combination of oxaliplatin, folinic acid, and 5-fluorouracil (5-FU), which was tested in the phase III ABC-06 trial (NCT01926236), as a second-line treatment for metastatic BTC [6]. Prior to the trial, Lamarca et al. conducted a systematic review of phase II trials, retrospective analyses, and case reports and found that there was insufficient evidence to recommend second-line chemotherapy in advanced BTC [7]. These available data highlight that therapeutic options after the failure of these two treatment lines are finite and not supported by prospective randomized clinical trials.

BTC is an aggressive malignancy that causes non-specific symptoms and thus is often diagnosed at advanced stages. Due to the late symptomatology, paucity of effective treatments, molecular diversity, and poor understanding of the complex molecular mechanisms and pathways, BTC has a dismal prognosis [8–11], with a poor median survival of 11.7 months despite therapeutic efforts [5].

The molecular diversity of BTC has led to the failure of most targeted therapies [11]. Nanoliposomal irinotecan (Nal-IRI) is a relatively new, highly stable liposomal nanocarrier encapsulated formulation of irinotecan [12], which is an inhibitor of topoisomerase-I that is converted to its metabolite SN-38 by carboxylesterase primarily in the liver; SN-38 is approximately 100 to 1000 times more potent than irinotecan [13]. The liposome serves as a spherical carrier vesicle for irinotecan that comprises a polyethylene glycol-containing bilayer membrane. Nal-IRI has several advantages including the protection of irinotecan from elimination in the blood stream, prolonged time in systemic circulation, and lower maximum plasma concentration to reduce drug-associated adverse effects. In addition, Nal-IRI can theoretically pass through the vascular pores in tumor tissues to increase intratumoral irinotecan levels. In preclinical settings, Nal-IRI at doses five times lower than those achieved with free irinotecan was shown to reach comparable local SN-38 levels within the tumor tissue, accompanied with superior antitumor activity [13, 14]. Moreover, both irinotecan and SN-38 exist in a pH-dependent equilibrium between an inactive carboxylate form and an active lactone form after intravenous injection. An acidic pH in the tumor microenvironment, such as that is present in BTC due to the hypovascularity and hypoxia, will promote the formation of the active lactone form. Thus, Nal-IRI may be able to tilt the pH-dependent balance toward the more active lactone form intratumorally to improve the antitumor activity of irinotecan [13].

The practice-changing phase III NAPOLI-1 trial investigated the effectiveness of Nal-IRI in combination with 5-fluorouracil (5-FU) and folinic acid (leucovorin) versus 5-FU and leucovorin in patients with pancreatic ductal adenocarcinoma (PDAC) who progressed after gemcitabine-based chemotherapy and reported that Nal-IRI extended overall survival (OS) and improved the objective response rate with a manageable safety profile [15]. Consequently, Nal-IRI was approved for use in these patients by the Federal Drug Administration (FDA) and the European Medicines Agency (EMA); it is currently not indicated for other diseases. There are similarities between PDAC and BTC, however, whether Nal-IRI may have a clinical benefit in BTC is unclear [16, 17].

In this retrospective, multicenter analysis, we assessed 14 patients with metastatic BTC who received Nal-IRI in combination with 5-FU and folinic acid. We determined the antitumor activity of Nal-IRI by assessing disease control rate (DCR), progression-free survival (PFS), and OS.

## Materials and methods

### Study design

This retrospective cohort study was conducted in accordance with the International Conference on Harmonization E6 Requirements for Good Clinical Practice and the ethical principles outlined in the Declaration of Helsinki.

The ethics committees waived the need for informed consent of the included patients for study conduction due to the retrospective nature of this analysis. However, all the patients had to provide informed consent before being treated with the off-label salvage therapy Nal-IRI in combination with 5-FU and folinic acid. Local authorities in Vienna approved the off-label use of Nal-IRI in combination with 5-FU and folinic acid. This study was designed by the Comprehensive Cancer Study Group of the Medical University of Vienna and conducted in collaboration with the Medical University of Innsbruck and the County Hospital in Vöcklabruck, Upper Austria. The Institutional Ethics Committees of the Medical Universities of Vienna and Innsbruck and Linz have approved this study (Number: 1131/2019).

### Patients

All patients who were eligible for this study had a histologically confirmed diagnosis of non-resectable and metastatic BTC (ICD-10 codes C22.1, C23, and C24), measurable disease according to the Response Evaluation Criteria in Solid Tumors classification version 1.1., and were treated with the salvage therapy regimen Nal-IRI in combination with 5-FU and folinic acid at the Division of Clinical Oncology at the Medical Universities of Vienna and Innsbruck and the County Hospital in Vöcklabruck between May 2016 and January 2019. Prior to May 2016 no metastatic BTC patient was treated with Nal-IRI in Austria. None of the metastatic BTC patients who were treated with Nal-IRI in combination with 5-FU and folinic acid were excluded. Other eligibility criteria at baseline included the following: Eastern Cooperative Oncology Group (ECOG) performance status score of 0–2; measured or calculated creatinine clearance of > 60 mL/min; adequate bone marrow function indicated by a minimum leukocyte count of 3 × 10^9^ cells/L, an absolute neutrophil count of 1.5 × 10^9^ cells/L, and a platelet count of 100 × 10^9^ cells/L; and adequate hepatic function with a total bilirubin up to 1.5 times the normal institutional upper limit.

### Treatment plan and toxicity assessment

The patients were treated with Nal-IRI in combination with 5-FU and folinic acid. Specifically, the patients received intravenous infusion of Nal-IRI at a dose of 80 mg/m^2^ (dose was calculated based on the free irinotecan base component) over 90 min, followed by intravenous folinic acid infusion at a dose of 400 mg/m^2^ over 30 min and intravenous 5-FU infusion at 2400 mg/m^2^ over 46 h, every 2 weeks. Toxicities were graded by the National Cancer Institute Common Terminology Criteria for adverse events version 4.0.

### Disease assessment

Objective response was assessed every 8–12 weeks or after six cycles of drug therapy using the response evaluation criteria in solid tumors (RECIST) 1.1 criteria. PFS was calculated from the date of registration to the date of first observation of progressive disease (PD), death due to any cause, or symptomatic deterioration. Patients who were alive and free of PD were censored on the last date of contact. The disease assessment was performed by the department of Radiology at the Medical University of Vienna, Medical University of Innsbruck, and County Hospital in Vöcklabruck.

### Statistical considerations

The data of the eligible patients were evaluated with descriptive statistics.

OS and PFS were analyzed using IBM SPSS Statistics software version 25 and presented using Kaplan–Meier curves. Data were presented using measures of central tendency, including means and medians, and frequency distributions were used to delineate the characteristics of the patients with metastatic BTC.

## Results

### Patient characteristics

Between May 2016 and January 2019, 14 patients, including 10 (71%) females and 4 (29%) males, received Nal-IRI therapy in combination with 5-FU and folinic acid. The clinical characteristics of the study cohort are summarized in Table 1. The median age at initial diagnosis was 59.3 years. The median age at initiation of the therapy Nal-IRI therapy in combination with 5-FU and folinic acid was 60.0 years. All patients had an ECOG performance status score between 0 and 1 and had metastatic lesions. 13 patients were diagnosed with intrahepatic cholangiocarcinoma and one patient was diagnosed with extrahepatic cholangiocarcinoma. For detailed characteristics of the patients, see Table 2.

**Table 1:** Treatment characteristics of advanced biliary tract cancer patients

Characteristics	Number of patients	Percentage
All patients	14	100
Female	10	71
Male	4	29
Median age at initial diagnosis	59.3	
Median age at Nal-IRI initiation	60.0	
ECOG performance status score		
0	11	79
1	3	21
TNM stage IVB	14	100
Biliary tract cancer subtype		
Intrahepatic cholangiocarcinoma	13	93
Extrahepatic cholangiocarcinoma	1	7
Nal-IRI treatment regimen		
Nal-IRI + 5-fluorouracil + folinic acid	14	100

**Table 2:** Detailed characteristics of the metastatic biliary tract cancer patients (*n* = 14)

Patients	Biliary tract cancer subtype	Age at initial diagnosis	Age at Nal-IRI initiation	Toxicity	Pre-treatment regimens in metastatic setting	Nal-IRI line	Therapy response
1. Female	Intrahepatic CCC	51.7	53.9	Diarrhea grade 2 Fatigue grade 1 Nausea grade 1 Oral mucositis grade 1	1st line: gemcitabine + cisplatin 2nd line: gemcitabine + nab-paclitaxel	3rd	PR
2. Female	Intrahepatic CCC	60.3	60.7	Nausea grade 1	1st line: gemcitabine + cisplatin	2nd	PR
3. Female	Intrahepatic CCC	78.8	79.3	Diarrhea grade 1 Neutropenia grade 3	1st line: gemcitabine + cisplatin	2nd	PR
4. Male	Intrahepatic CCC	54.5	54.9	Diarrhea grade 1	1st line: gemcitabine + cisplatin	2nd	PR
5. Male	Intrahepatic CCC	70.6	73.6	Diarrhea grade 1 Fatigue grade 1 Nausea grade 1	1st line: gemcitabine + cisplatin 2nd line: capecitabine + nab-paclitaxel	3rd	PR
6. Male	Extrahepatic CCC	73.5	74.1	No toxicities reported	1st line: gemcitabine + nab-paclitaxel	2nd	PR
7. Female	Intrahepatic CCC	32.7	43.6	Neutropenia grade 1 Thrombopenia grade 1	1st line: gemcitabine + cisplatin 2nd line: capecitabine + irinotecan 3rd line: capecitabine + nab-paclitaxel	4th	SD
8. Female	Intrahepatic CCC	64.0	64.8	Fatigue grade 2	1st line: gemcitabine + oxaliplatin 2nd line: capecitabine + nab-paclitaxel	3rd	PD
9. Female	Intrahepatic CCC	54.9	57.7	Neutropenia grade 1	1st line: gemcitabine + nab-paclitaxel 2nd line: capecitabine + oxaliplatin 3rd line: regorafenib 4th line: nintedanib	5th	PD
10. Female	Intrahepatic CCC	60.4	60.9	Diarrhea grade 1	1st line: gemcitabine + cisplatin	2nd	PD
11. Female	Intrahepatic CCC	75.9	77.5	Anemia grade 1	1st line: Gemcitabine + Cisplatin 2nd line: 5-fluorouracil + folinic acid 3rd line: gemcitabine + nab-paclitaxel	4th	PD
12. Female	Intrahepatic CCC	53.5	54.6	Neutropenia grade 3	1st line: gemcitabine + cisplatin 2nd line: gemcitabine + nab-paclitaxel	3rd	PD
13. Female	Intrahepatic CCC	56.4	57.3	Nausea grade 1 Anemia grade 1	1st line: gemcitabine + cisplatin	2nd	PD
14. Male	Intrahepatic CCC	58.3	59.4	Nausea grade 2 Oral mucositis grade 1	1st line: gemcitabine + cisplatin 2nd line: capecitabine + oxaliplatin	3rd	PD

### Treatment plans

Eleven of the 14 patients were administered gemcitabine and cisplatin as the first-line treatment. Additionally, two patients were administered gemcitabine and nab-paclitaxel as the first-line treatment and one patient received gemcitabine in combination with oxaliplatin (Table 1). Nal-IRI was administered in combination with 5-FU and folinic acid as second-, third-, fourth-, and fifth-line treatment in 6 (43%), 5 (36%), 2 (14%), and 1 (7%) patient, respectively.

### Treatment-associated toxicities

Among the 14 patients, diarrhea and nausea were documented in 5 (38%) patients, whereas neutropenia was observed in 4 (30%) patients. Other toxicities described during the observation time were fatigue, oral mucositis, anemia, and thrombocytopenia. Except for grade 3 neutropenia observed in 2 (14%) patients, all side effects were mild (grade 1 or 2, Table 3).

**Table 3:** Adverse events observed during Nal-IRI treatment in combination with 5-FU and folinic acid

Toxicity	Number of patients	Percentage
Diarrhea	5	38
Grade 1	4	
Grade 2	1	
Nausea	5	38
Grade 1	4	
Grade 2	1	
Fatigue	3	23
Grade 1	2	
Grade 2	1	
Oral mucositis	2	15
Grade 1	2	
Thrombocytopenia	1	8
Grade 1	1	
Neutropenia	4	31
Grade 1	2	
Grade 2	-	
Grade 3	2	
Anemia	2	15
Grade 1	2	

### Clinical efficacy

**Fig. 1:**
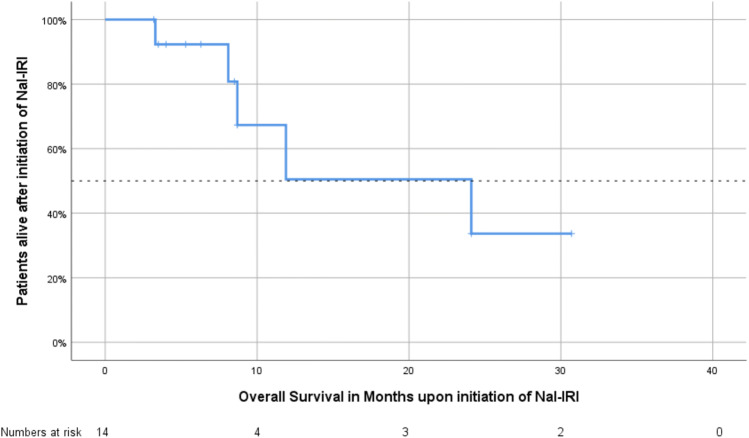
Kaplan–Meier estimates of overall survival in patients with metastatic biliary tract cancer following the initiation of Nal-IRI treatment

**Fig. 2:**
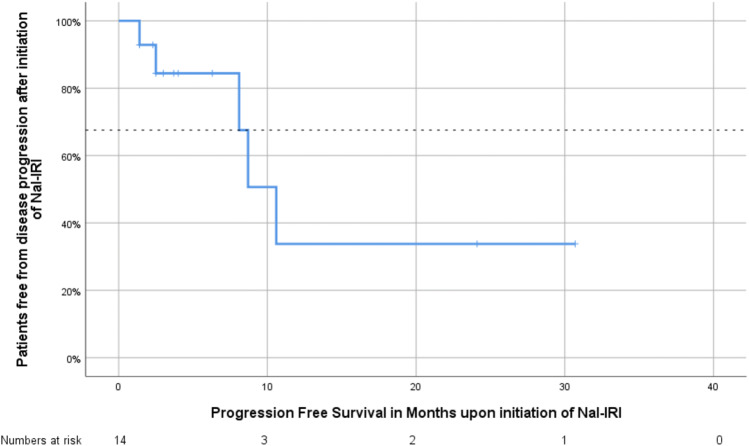
Kaplan–Meier estimates of progression-free survival in patients with metastatic biliary tract cancer following the initiation of Nal-IRI treatment

All 14 patients were eligible for the analyses of response. Six patients achieved partial response (PR), and one patient achieved stable disease (SD); therefore, the DCR was 50% (Table 4). The median time of OS after the diagnosis of metastatic disease was 35.7 months (95% confidence interval 20.7–47.5 months), whereas nine patients were alive at the date of censoring (January 2019). The median OS after the initiation of Nal-IRI treatment was 24.1 months (95% confidence interval 7.4–41.0 months, Fig. 1), and the median PFS after the initiation of Nal-IRI treatment was 10.6 months (95% confidence interval 7.9–13.3 months, Fig. 2, Table 5).

**Table 4:** Tumor response to Nal-IRI treatment in combination with 5-FU and folinic acid

Therapy response	Number of patients	Percentage
PR	6	43
SD	1	7
PD	7	50
DCR	7	50
ORR	6	43

**Table 5:** Progression-free survival since Nal-IRI treatment (upper panel) and overall survival since Nal-IRI treatment (lower panel)

Survival rate	Months (95% confidence interval)
PFS (since Nal-IRI initiation)	
Mean	15.3 (6.9–23.8)
Median	10.6 (7.9–13.3)
OS (since Nal-IRI initiation)	
Mean	18.7 (11.4–26.1)
Median	24.1 (7.4–40.8)

## Discussion

In the current study, we provide the first evidence of the antitumor activity of Nal-IRI in combination with 5-FU and folinic acid in 14 patients with advanced and metastatic BTC after failure of the first-line gemcitabine-based chemotherapy regimen. Despite advanced disease and prior treatment, Nal-IRI achieved a DCR of 50%, a median PFS of 10.6 months, and a median OS of 24.1 months. These results provide evidence for the antitumor activity of Nal-IRI in metastatic BTC. As a comparison, cisplatin in combination with gemcitabine as the first-line therapy achieved a median PFS of 8.0 months and a median OS of 11.7 months in the phase III ABC-02 trial. Conversely, the phase III ABC-06 trial recently achieved a median OS of 6.2 months with the second-line therapy regimen including 5-FU, folinic acid, and oxaliplatin. Further, several phase II clinical trials studied the efficacy of conventional irinotecan as a single-agent or in combination with other agents in advanced BTC; however, conventional irinotecan exhibited only a modest clinical activity in these trials [18–21]. One reason for the high response rates observed in our analysis may be due to the unique features and advantages of Nal-IRI including the protection of irinotecan from elimination in the blood stream, prolonged time in systemic circulation, lower maximum plasma concentration and increased antitumoral activity in the acidic tumor microenvironment of biliary tract cancer.

These encouraging data and, in particular, the possible use of Nal-IRI in combination with 5-FU and folinic acid as induction chemotherapy should be evaluated in further well-designed clinical trials.

Similar to BTC, PDAC has a poor prognosis and is resistant to many therapeutic approaches. Due to the heterogeneity and complexity of PDAC, most targeted agents failed to demonstrate improvement in the OS. However, in the practice-changing NAPOLI-1 trial, Nal-IRI had significant clinical benefit for patients who progressed on gemcitabine-based therapy [15]. In that study, the median OS in the patients treated with Nal-IRI in combination with 5-FU and folinic acid was 6.1 months, which was significantly better that the median OS of 4.2 months in the group treated with 5-FU and folinic acid (hazard ratio 0.67, 95% confidence interval 0.49–0.92, *p* = 0.012). Moreover, Nal-IRI had a manageable safety profile.

Currently, Nal-IRI is being tested in over 30 clinical trials in different disease entities and settings, including head and neck malignancies, brain metastasis in breast cancer, neuroendocrine cancer, and colorectal cancer. Of particular interest are four prospective trials that are currently recruiting patients to evaluate Nal-IRI in BTC. In the phase II randomized trial NALIRICC (NCT03043547), Nal-IRI in combination with 5-FU and leucovorin is compared with 5-FU and leucovorin. In another phase I/II trial (NCT03337087), patients are assigned to the therapy regimen including Nal-IRI, 5-FU, leucovorin, and rucaparib. Another important phase II trial, NIFTY (NCT03524508) is recruiting over 170 patients with metastatic BTC to evaluate the treatment regimen assessed in the NAPOLI-1 trial for PDAC. Finally, the randomized multicenter phase II trial NIFE (NCT03044587) is allocating patients to receive Nal-IRI in combination with 5-FU and leucovorin or cisplatin and gemcitabine.

This study has several limitations. It was a non-randomized and retrospective analysis of a multicenter registry. The study cohort was small and lacked an adequate control group.

Further, the cohort is skewed to young age and is dominated by female patients. Moreover, the disease assessment was performed by the local departments of Radiology and not by a blinded central review.

It is important to stress that this analysis may contain survivorship bias since it was based on the data of patients who had already received a median of 2 prior treatments and experienced a relatively long median OS of 35.7 months.

Yet, this is the first study describing the antitumor activity and the potential clinical benefit of Nal-IRI as a later treatment line in metastatic BTC. Thus, Nal-IRI should be considered as a viable therapy alternative in biliary tract cancer. However, further studies and clinical trials are warranted to understand the complex tumor biology and improve OS in BTC.
